# A client-centered relational framework on barriers to the integration of HIV and substance use services: a systematic review

**DOI:** 10.1186/s12954-019-0347-x

**Published:** 2019-12-19

**Authors:** Rogério Meireles Pinto, Yun Chen, Sunggeun ( Ethan) Park

**Affiliations:** School of Social Work, University of Michigan—Ann Arbor, 1080 South University Avenue, Ann Arbor, MI 48109 USA

**Keywords:** HIV, Substance use, Service integration, Barriers, Client-centered relational framework

## Abstract

**Background:**

Given the close connection between human immunodeficiency virus (HIV) infection and substance use disorder (SUD), access to integrated HIV and SUD services is critical for individuals experiencing both challenges and their biopsychosocial conditions.

**Method:**

Adopting an integrative method, this systematic review included 23 empirical studies published between 2000 and 2018. Articles investigated providers’ and clients’ perspectives on barriers to accessing integrated HIV and SUD services in various service settings (e.g., HIV primary care, SUD treatment, pharmacy).

**Results:**

Using a client-centered relational framework, we identified barriers in three relational domains with “the client” as the focus of each: client-provider, client-organization, and client-system. The review shows that (1) barriers to HIV and SUD services do not exist in isolation, but in the dynamics within and across three relational domains; (2) service providers and clients often have different perceptions about what constitutes a barrier and the origin of such barriers; and (3) interprofessional and interorganizational collaborations are crucial for integrating HIV and SUD services.

**Conclusion:**

This review points out the limitations of the conventional paradigm grouping barriers to service integration into isolated domains (client, provider, organization, or system). Reforms in service arrangements and provider training are recommended to address barriers to integrated services.

## Background

Substance use disorders (SUDs) are common among people living with HIV (PLWH). People who inject drugs (PWID) or have SUDs are more likely to be exposed to and become infected by HIV and experience a more accelerated HIV progression [1–5]. Even for those individuals for whom substance use is not the transmission route for HIV, the pervasive stigma experienced by PLWH sometimes results in substance use as a coping strategy [6]. Given this close relationship between HIV and SUD, it is critical that both prevention and treatment services aiming to address the two conditions be integrated to achieve optimal health outcomes [7, 8]. A robust collection of literature exists on the integration of HIV and SUD treatment services, which focuses on describing the advantages of myriad services combinations, such as SUD counseling in HIV medical care, HIV testing in SUD outreach services, or introducing pre-exposure prophylaxis (PrEP) in SUD treatment [9–12]. Regrettably, the literature also shows that people with HIV and SUDs face myriad barriers accessing and using either or both HIV and SUD services [13, 14]. These barriers affect and, in turn, are affected by how service consumers (“clients”) interact with service providers (e.g., mutual distrust between providers and clients), with the organizations where they receive services (e.g., distant location, lack of transportation), and with the social systems (e.g., stigmatization and disenfranchisement) they navigate in order to find the HIV and SUD services they need [15–17].

Many studies draw from the service providers’ or clients’ data to identify barriers at the interpersonal, organizational, and system/structural levels. However, the interpretation of barriers to HIV or SUD services as isolated entities at different unrelated domains may obscure the fact that barriers are relational and exist in the intersection of client and provider, client and organization, and client and systems. Using a relational approach to guide our systematic review, we identified barriers in each of those intersections, which are more specific to the difficulties that clients face when trying to navigate multiple domains of services in order to address their HIV and SUD needs. Factors that hinder access to these services have been identified, and effective integrated interventions have been recommended [18–21]; however, to the best of our knowledge, little effort has been made to understand the multilayered factors that may hinder service access and utilization from a relational lens.

This integrative review includes 23 articles published between 2000 and 2018 which investigated providers’ and clients’ perspectives on HIV and SUD service barriers in various service settings (e.g., primary care, HIV clinics) in the U.S. As practitioners and researchers on HIV and SUD, our emphasis here is on service integration, and not one or another type of service—we aimed instead to address the important-yet-understudied issue of integration. While acknowledging the merits of a broader approach looking at specific intervention models, our review is designed to provide more specificity and depth; it thus helps to narrow an important gap in the literature, as follows. The review provides evidence supporting our assumption that barriers to HIV and SUD services do not exist in isolation; rather, these barriers are interrelated and thus inform how clients relate to their service providers and healthcare systems. This review also highlights how service providers and clients often have different perceptions about the definition and source of barriers to healthcare. Based on our findings, we provide recommendations, specifically regarding the importance of person-centered approach and interprofessional and interorganizational collaborations, for future research and for improving best practices.

### Relational conceptual framework

**Fig. 1:**
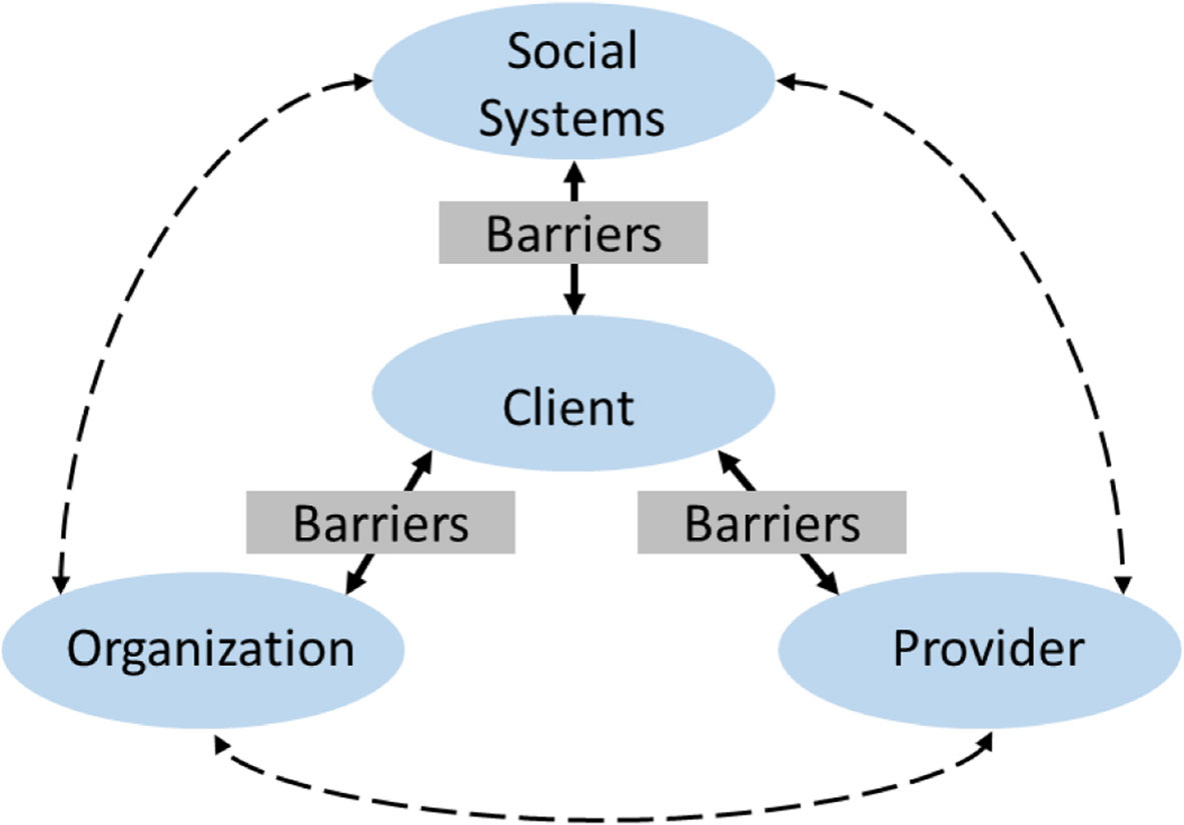
Barriers to service integration—a client-centered relational framework. Guided by a client-centered relational approach, this study directs focus to the relationships between clients and their providers, clients and service organizations, and clients and the socioeconomic structures under which they live. Barriers residing in these relationships affect the dynamic and ecological interactions when clients enter a service system

Both the ecological and systems theories [22, 23] suggest that a myriad of inter-connected barriers to access and delivery of biopsychosocial services exist for clients who are seeking care. Figure 1 illustrates the domains within which these barriers can be found: client, organizational (including providers), and structural/system levels. However, many barriers to access and delivery (for example, housing instability) have been conceptualized as a client-level issue when, in fact, housing is a structural issue affecting millions of people in the U.S. [24]. Similarly, given the popularity of the rational patient model, other structural-level (e.g., job insecurity and limited service availability) and organizational-level (e.g., long waitlist, lacking flexible service hours) barriers have been conceptualized as client-level issues (e.g., chaotic and unhealthy lifestyles, noncompliance), manifested in the common perceptions of clients’ lacking ability or will to making rational decisions on their health and social care [25–27]. This fails to describe the intricate and relational nature of the barriers impeding clients from accessing health and social services. For this systematic review, we were guided by a relational approach that directs focus to the relationship between clients and their providers and service organizations, and to the socioeconomic structures under which they live.

Clients have direct and indirect relationships with providers (in organizations), with organizations (within systems), and with social structures and systems. Social, economic, and political environments may introduce multiple barriers to access and delivery of HIV and SUD services. Growing evidence on how social and physical environments contribute to health inequality emphasizes the importance of understanding access/delivery from a relational lens [28]. This relational lens allows us to imagine barriers to access (e.g., poverty, lack of social support/capital), usually attributed to clients, as structural inequalities (e.g., inadequate safety nets, residential segregation, and mass incarceration minorities). Misaligned interests between clients and organizations also influence access/delivery of HIV and SUD services. For example, resource-deprived organizations may prioritize resource acquisition over services and programs addressing clients’ access, such as discontinuing less profitable services or laying off specialized workforce rather than expanding service lines to address both HIV and SUD issues or hiring specialized care coordinators [29, 30].

Mismatched or contentious relationships between clients and providers often follow from front-line service provider’s position to simultaneously address clients’ multifaceted service needs, providers’ limited capacity and resources, and incentives and rules imposed by organizations and institutions [31, 32]. For example, lack of staff training on how to work with co-morbid conditions (e.g., HIV and SUD) may affect negatively how providers work with such clients or encourage providers to allocate limited resources for addressing simpler cases that yield greater impacts—resulting in unresponsive and disrespectful care experiences for clients. Resource-deprived and unexperienced providers may make care decisions based on scientific evidence around provider-driven care, rather than engaging clients into care decisions and adjusting interventions to their circumstances and preferences—i.e., patient-centered care [31, 32]. Due to information gaps and possibly client’s negative attitudes toward unresponsive care systems, a client’s repeated non-compliance to a prescribed plan, perhaps justifiably due to lack of resources, may confirm providers’ beliefs and stereotypes about that client—further deepening mutual distrust and hindering the client’s access to life-saving health and social services (Park ES: Beyond patient-centred care: a conceptual framework of co-production mechanisms with vulnerable groups in health and social service settings, forthcoming).

## Methods

This systematic review is grounded in the relational framework above and guided by an integrative review method [33] including specification of the purpose of the review, search of the extant literature and evaluation of primary sources, specification of an analytical strategy, and presentation of findings in a practical and user-friendly manner.

### Literature search procedures

We conducted literature searches on EBSCO*host* and Web of Science through the University of Michigan’s library for the years 2000–2018. Acknowledging the fast developments in intervention science in the field of harm reduction—e.g., broad implementation of HIV prevention pre-exposure prophylaxis—we restricted our review to the past two decades, in order to exclude interventions that are outdated while capturing the most contemporary trends in service delivery. Recognizing that some articles in PubMed might not be included in either EBSCO*host* or Web of Science, we also searched PubMed in a separate search using the same terms and following the same screening procedures. To improve the likelihood of finding articles about barriers to services at the intersection of the two fields, we searched for articles about HIV and SUD services separately *and* together, using the following permutations:

- *Abstract (in EBSCOhost) and topic (in Web of Science)*: “substance abuse” OR “substance use” OR “substance misuse” OR “drug addiction” OR “drug abuse” OR “drug use” OR “drug users” OR “substance users.”
- *Abstract (in EBSCOhost) and topic (in Web of Science)*: “HIV prevention” OR “PrEP” OR “antiretroviral” OR “Pre-Exposure Prophylaxis” OR “HIV treatment” OR “PLWH.”
- *Abstract (in EBSCOhost) and topic (in Web of Science)*: “service” OR “treatment” OR “intervention” OR “care.”
- *Abstract (in EBSCOhost) and topic (in Web of Science)*: “access” OR “utilization” OR “adherence.”
- *Title or abstract (in EBSCOhost), and title or topic (in Web of Science)*: “barriers.”

The search terms (a) and (b) were each combined with (c), (d), and (e) as two separate searches for barriers to HIV and SUD services, respectively, as well as together, combined with (c), (d), and (e) for barriers at the intersection of the two fields. We searched for the term “barriers” in the title for both databases in the HIV and SUD separate searches and the abstract (EBSCOhost), or as a topic (Web of Science) in the HIV/SUD combined search.

We used the online platform Rayyan QCRI (https://rayyan.qcri.org/) [34] for managing the initial search results, including removing duplicates, removing articles that clearly fell beyond the scope of our review, and conducting the title and abstract screening of all articles that fulfilled our initial inclusion criteria. This online platform allowed two of our collaborators to blind review all the titles and abstracts independently.

### Inclusion and exclusion criteria

In the initial screening, we included peer-reviewed studies on the barriers to service access in the intersection of HIV prevention and care and SUD services in the United States (U.S.). We excluded papers that were (1) not about the U.S., (2) not about the intersection of the two fields, (3) reviews or commentary articles, and (4) clinical trials or evaluations of particular service programs. After this initial round of titles and abstract screening, the three authors held multiple face-to-face discussions and agreed to exclude studies that were (5) only on the adherence to treatment or medication of one specific substance. Since our review focuses on service practices, we also excluded studies that (6) analyze administrative or policy documents. In order to be readily able to use the review’s findings to guide practice and policy development, we focused the search on the U.S. because different countries have different healthcare and social service systems and thus myriad different barriers.

### Article selection procedures

**Fig. 2:**
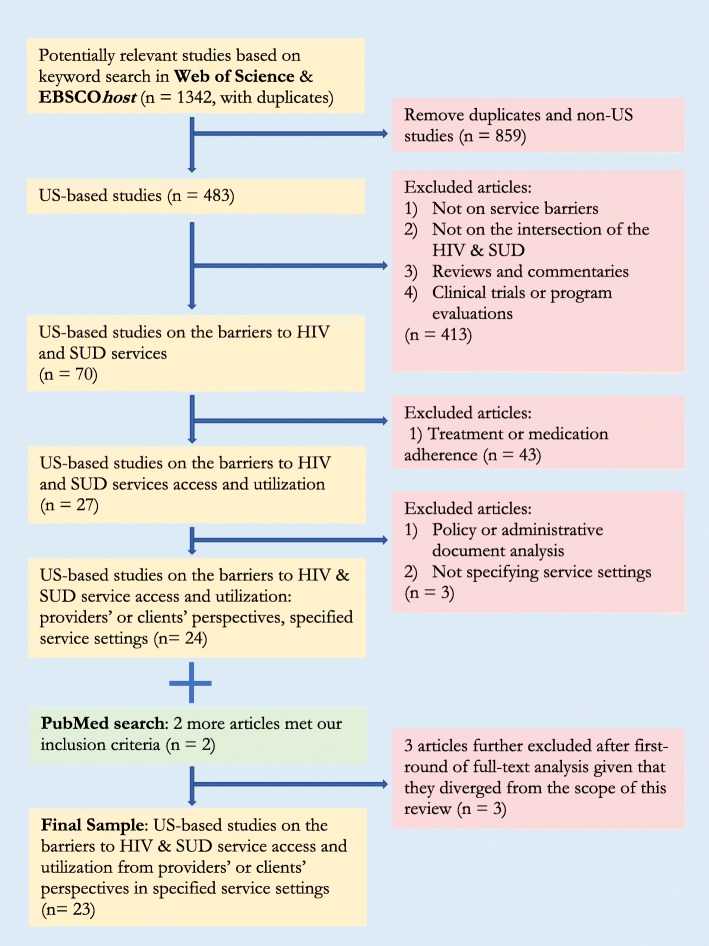
Summary of article selection and inclusion/exclusion criteria. Based on a comprehensive search and screening of literature in Web of Science, EBSCO*host*, and PubMed, the study identified 23 articles that empirically examined the barriers to HIV and SUD service access from the perspectives of providers and clients in myriad service settings in the U.S.

Figure 2 summarizes the article selection procedures. Our initial search of EBSCO*host* and Web of Science yielded 483 U.S.-based articles. Using our inclusion/exclusion criteria, on our first-round title and abstract screening, we excluded 413 papers. Then, after the initial screening, we agreed to exclude studies that exclusively focused on adherence since it entails questions characteristically different from those of service access and utilization. This left us with 27 articles to consider for further review. We did an initial categorization of the 27 articles based on title, abstract, and method, and screened out three (two policy/administrative analysis and one without specific service setting information) (*n* = 24). Following the same searching and screening procedure, the additional search on PubMed yielded two qualifying articles, resulting in a total of 26 articles for initial full-text coding. After the initial coding of these 26 articles, we further identified three papers that we agreed to exclude because they turned out to be outside the scope of the review. Two of the three articles did not center on barriers to service access and utilization [35, 36], and one article solely focused on client’s individual-level barriers (e.g., denial of HIV diagnosis, forgetting medication, or prioritizing drug use over HIV treatment) [5]. The final selection included 23 articles that empirically examined the barriers to HIV and SUD service access from the perspectives of providers and clients in myriad service settings in the U.S.

### Article analysis

Guided by an integrative review method [33], we used a published data analysis plan developed by the Pinto and colleagues [37], which included purposive sampling, a modified version of grounded theory, and collaborative interpretation [38]. “Purposive sampling” here is used to describe the above procedures we used to select the articles for this review—specific search terms and inclusion and exclusion selection criteria. Our analysis reflects a modified version of the grounded theory [39] in that we selected the final set of articles grounded in our experiences as researchers and practitioners who have provided both HIV and SUD services. We used a collaborative approach, whereas each chosen article was examined independently by at least two of the authors and discussed by all three. Our lengthy (average 2 h) and frequent (average weekly) discussions were used to address researcher bias and to reach 100% agreement on selected papers and the final results (reflected in Table 1). Our analysis consisted of the following steps:

- Step 1—We extracted all information related to service barriers from each article.
- Step 2a—We categorized barriers at the interpersonal, organizational, and system levels.
- Step 2b—We examined each barrier selected from step 2a and held discussions on how to reconceptualize them grounded in our relational framework.
- Step 3—We categorized all barriers to reflect a client-centered relational framework highlighting the dynamic relationships between clients *and* service providers, organizations, and social systems.

**Table 1:** Barriers to integrating HIV and SUD services for clients with multiple biopsychological and socioeconomic issues

Articles	Sample	Client-provider domain	Client-organization domain	Client-system domain
**Primary care setting**				
Turner et al. [40]	Clients	♦ Concerns about drug users’ lacking adherence to ART or HAART ♦ Biases toward former drug users		
Lucas et al. [41]	Clients		♦ Inability to keep scheduled clients ♦ Emergency care services overload ♦ Distrust of medical establishment	♦ Unstable life conditions ♦ Social isolation
Loughlin et al. [42]	Providers	♦ Negative attitudes toward clients’ appointment truancy and HIV denial ♦ Concerns about HIV medication and drug use interactions		♦ Homelessness ♦ Social isolation ♦ Unstable life conditions
Ding et al. [43]	Clients and providers	♦ Lack experiences in treating IDUs ♦ Bias towards HIV-infected IDUs’ ♦ Ability to adhere to treatment ♦ Provider afflicted by depression and stress	♦ High caseload	
Turner et al. [44]	Providers	♦ Lack of knowledge about the efficacy of methadone in HIV prevention	♦ Lack of familiarity with clients who misuse narcotics due to low volume ♦ Lack of social workers, counselors, psychiatrists, and psychologists ♦ Burdensome paperwork ♦ Lack of HIV specialty services	♦ Inadequate financial reimbursement
Ware et al. [45]	Clients	♦ Bias towards treatment adherence ♦ Lack of awareness of the stabilizing role of HAART medication		♦ Stigma about HIV-positive drug users ♦ Unstable life conditions
Vaughn [46]	Provider	♦ Bias toward clients: - Denial of medication effectiveness - Distrust of the medical establishment - Beliefs about HIV infection as a man-made form of genocide - Difficulties controlling substance use due to pain management - Mental health conditions	♦ Long waitlist for substance use disorder treatment ♦ Lack of interprofessional collaboration ♦ Lack of pain management therapies ♦ Negative experiences with the medical establishment	♦ Disenfranchisement ♦ Lack of insurance for substance use disorder treatments
Cunningham et al. [47]	Providers	♦ Lack of confidence/knowledge to address substance misuse, drug interaction, and substance misuse treatment approaches	♦ Lack of collaboration across service providers in different health systems	♦ Shortage of licensed buprenorphine prescribers
Macalino et al. [48]	Providers	♦ Lack of knowledge and negative attitudes toward syringe sharing, exchange programs, distribution practices, and syringe prescription as HIV prevention	♦ Lack of familiarity with IDUs	♦ Pharmacist stigma against IDUs ♦ Police stigma against IDUs
Westergaard et al. [49]	Providers	♦ Discomfort treating IDUs with HIV ♦ Bias toward IDUs’ inability to adhere to HAART	♦ Lack of HIV or SUD services ♦ Lack of experts in HIV care ♦ High caseload ♦ Lack of familiarity with HIV clients	
Gwadz et al. [50]	Clients	♦ Poor provider-client communication ♦ Distrust between provider and client ♦ Stigmatization of and minimization of clients’ life experiences		♦ Racism ♦ Poverty ♦ Lack of social support ♦ Housing instability ♦ Lack of public assistance
Campbell et al. [51]	Providers	♦ Provider lacking flexibility and creativity in decision-making ♦ Poor provider-client communication ♦ Provider negative attitudes towards clients’ substance use	♦ Bureaucratic barriers (e.g., paperwork) ♦ Lack of and poor quality of outreach services ♦ Lack of coordinating staff ♦ Lack of referral tracking and evaluation system	♦ Housing instability ♦ Illicit drug use
**Substance use disorder (SUD) treatment setting**				
Whetten et al. [52]	Clients			♦ Lack of transportation and inaccessible service resources
Spector et al. [10]	Providers	♦ Lack of knowledge and bias toward: - Clients’ ability to PrEP adherence - “Risk compensation” - PrEP side effects - PrEP reimbursement ♦ Lack of training in psycho-pharmacology	♦ Lack of structure for referral making ♦ Lack of staff to prescribe PrEP and to monitor adherence	
Shrestha et al. [53]	Clients		♦ Irregular access to PrEP refills or follow-up visits	♦ High PrEP medication cost ♦ Lack of insurance coverage ♦ Stigma and social discrimination
Roth et al. [54]	Clients		♦ Lack of PrEP education access	♦ Lack of access to primary care and thus HIV risk assessment exposure and PrEP education
**Multiple settings**				
Downing et al. [55]	Providers		♦ Lack of flexible testing hours ♦ Distrust of organization’s treatment of confidential information ♦ Lack of training for providers ♦ Inadequate collaborations across agencies	♦ Distrust of government’s treatment of confidential information
Wyatt et al. [56]	Clients	♦ Poor provider-client relationship and distrust	♦ Long wait lists and difficulty obtaining an appointment ♦ Distrust of organization’s treatment of confidential information	♦ Financial problems
Knowlton et al. [57]	Clients	♦ Poor provider-client communication	♦ Lack of outpatient services	♦ Lack of social support ♦ Housing instability ♦ Racism
Wright et al. [58]	Providers		♦ Lack of capacity to provide HIV testing ♦ Additional administrative or clinical burdens coming with service integration ♦ Poor inter- and intra-agency communication	♦ Lack of political support for HIV test (low awareness, funding, priority) ♦ Lack of regulations addressing service integration ♦ Lack of transportation ♦ Lacks state funding and infrastructure supporting service integration
Biello et al. [59]	Clients and providers	♦ Poor provider-client relationship and distrust ♦ Low willingness to prescribe PrEP to drug users	♦ Burdensome PrEP screening and retention protocols ♦ Lack of professional and interagency collaboration	♦ HIV-related stigma ♦ Poor regional infrastructure and capacity for PrEP delivery ♦ Housing instability ♦ Poverty ♦ Disenfranchisement ♦ Transportation difficulties
**Other settings**				
Schoeneberger et al. [60] (NIDA Cooperative Agreement in Kentucky)	Clients		♦ Long waitlist ♦ Some programs do not take women or women with children	♦ Poverty ♦ Lack of regional substance use treatment resources ♦ Gender bias in treatment eligibility criteria
Lutnick et al. [61] (pharmacy)	Clients	♦ Stigmatization of clients’ behaviors	♦ Lack of private space for clients to dispose of syringes and receive services ♦ Distrust of organization’s treatment of confidential information	

## Results

Table 1 shows our final set of 23 articles, which includes empirical studies on barriers to integration of HIV and SUD services, including data from service providers (*n* = 10), clients (*n* = 11), and both clients and providers (*n* = 2). Thirteen of the 23 articles used quantitative methods or secondary survey data analysis, nine used qualitative interviews or case studies, and one used a mixed-methods approach. Twelve studies were conducted in HIV primary care settings, four in SUD treatment settings, five drew samples from multiple settings including healthcare clinics and community-based organizations, and two from “other settings.”

The reviewed articles highlight service integration aimed to help individuals facing myriad challenges related to both HIV exposure and SUD. Several studies recommended the integration of services in HIV primary care settings for clients who also inject drugs. Proposed modalities of service integration included having primary care providers distribute sterile syringes [48], providing methadone and buprenorphine treatments [44, 47], strengthening clients’ social supports [57], and addressing sociodemographic obstacles to care and competing health needs [42]. Regarding service integration in SUD treatment settings, this review highlights the need for medical treatment access for HIV-positive injection drug users receiving services in community-based settings [56, 57]. The review also highlights the need for integrating preventive services such as HIV testing [58] and pre-exposure prophylaxis (PrEP) [10, 53, 54, 59] into community-based facilities. This would improve service availability, accessibility, and connectivity for SUD clients who are exposed to environments where HIV is prevalent.

Table 1 reveals common themes that emerged from selected articles regarding barriers to service within three domains of reference. Below, we present a summary of barriers in each domain to highlight unique client-centered dynamic relationships with service providers, organizations, and social systems, reflecting the conceptual frame provided above.

### Client-provider relational domain

Barriers under this domain were grouped as (a) provider concerns or biases toward clients, (b) provider lacking competencies, and (c) poor provider-client relationship. Many articles investigating the client’s perspective identified providers’ biases and concerns about clients’ characteristics—for example, clients’ difficulties in adhering to medical treatments, lack of readiness to initiate treatment, denial of HIV status, or inability to keep appointments [40, 42, 43, 45, 46, 49–51, 61]. Another type of client-provider barrier was providers’ lack of awareness, knowledge, skills, experience, or confidence in serving clients diagnosed with co-morbidities and/or in carrying out specific treatment models [10, 43, 44, 47, 48, 51]. Poor provider-client relationships, including poor communication, confidentiality issues, and mutual distrust, were identified in many articles [46, 50, 51, 56, 57, 59]. One study also pointed out providers’ mental health status and stressful life conditions as a potential barrier [43], and thus highlighted the need of self-care among providers for carrying out services effectively.

### Client-organization relational domain

Barriers under this domain were grouped based on organizational issues: (a) inconvenient procedures for clients; (b) challenges for providers, such as lack of service experience and competency; and (c) lack of interprofessional and/or interagency collaborations. These clinical and administrative issues may cause difficulties for both clients to access and for providers to deliver services. Examples include inflexible HIV testing hours [55], long waitlists [46, 56, 60], difficulty in obtaining appointments [56], and lack of regular medication refills or follow-up visit arrangements [53]. Our review identified the following key barriers within the client-organizational domain: lack of training resources or low training quality [54, 55], high caseload [43, 49], and burdensome paperwork [44, 51, 59]. Other key barriers include lack of access to consultants and services from other professions [10, 44, 46, 47, 59], ineffective interagency referral structure [10, 51], and poor inter- and intra-agency communication [51, 58]. This review found that some HIV clinics were in areas with a relatively lower volume of SUD clients; thus, providers in these clinics had limited knowledge and experiences of working with dual-diagnosis clients [44, 48, 49].

### Client-system relational domain

Barriers under this domain were grouped based on structural issues that lead to (a) socioeconomic and health-related disparities for clients, (b) deficiencies in resources and social support available to clients, and (c) stigmatization and discrimination of clients’ behaviors and socioeconomic conditions. The review found that individuals with both HIV and SUDs may face poverty, homelessness, social instability, and an unfair criminal justice system [41, 42, 50, 51, 59, 60]. Collectively, these conditions contribute to further barriers, such as multiplication of competing demands [10, 45, 56], and are particularly related to late access of HIV and/or SUD treatment [49]. Deficiencies in resources and supports include insurance coverage, transportation, public assistance, treatment options, and social support [46, 50, 52–54, 56–60]. Unequal treatment and inequitable resource distribution [44, 47, 58], such as insufficient financial reimbursements [44], directly affect individuals diagnosed with co-morbidities like HIV and substance use disorders. Moreover, individuals with both HIV and SUD also face social, economic, and political stigmatization and discrimination in the form of racism, gender biases, and stereotypes [45, 48, 50, 53, 57, 59, 60]. Structural stigmatization and discrimination may lead to negative experiences in service systems [46, 59] and, even worse, may block initial entry to service systems [42, 50, 60].

## Discussion

This review of 23 empirical studies identified a myriad of barriers to accessing integrated HIV and SUD services in different healthcare settings. We grounded our analysis of selected papers in a relational framework suggesting that barriers can be best understood and therefore addressed by linking them to the relationships that exist between clients and providers, clients and organizations, and clients and systems. This perspective shifts the origin of access barriers to where they belong. For example, housing instability is an access barrier often discussed as a client’s problem (e.g., lack of work ethic). Alternatively, homelessness can be understood as a conflict between clients and socio-economic and political structures. As many clients with HIV and SUD issues face difficulties in finding stable jobs and income sources, they are more likely to experience housing instability, which in turn creates further barriers to employment to access to HIV and SUD treatments. From a relational perspective, such service barriers can be better understood as conflicts between client and structures requiring macro-level policy and program interventions, rather than issues pertaining exclusively to the clients (e.g., lack of will or capacity) or providers (e.g., lack of respect for clients).

We note that 21 out of the 23 reviewed articles reported barriers in more than one domain and eight articles reported barriers in all three domains. This finding illustrates that barriers to the access and delivery of integrated HIV and SUD services should not be treated as isolated issues pertaining solely to clients, providers, organizations, or systems. Rather, barriers in one domain often co-exist with barriers in the other domains. For example, in the case study by Vaughn [46], the unequal distribution of resources (e.g., certain populations were disenfranchised from basic social and medical establishments) and its negative impacts on the clinical and administrative operations of service organizations (e.g., long waitlist, limited eligibility criteria, and the lack of interprofessional services) directly contributed to the disadvantaged conditions experienced by the clients and to the negative relationships between clients and service providers. Another example from Campbell and colleagues [51] study showed that the unstable life conditions and complex challenges faced by clients with dual diagnoses required providers to be more aware of and knowledgeable about the need for and the mechanisms of integrating HIV and SUD treatment services. However, providers’ awareness and ability to integrate services may be hindered by organizational factors, such as the lack of coordinating staff, interagency collaboration, and burdensome administrative tasks. The relational approach highlights the need for treating barriers, identified at one level or another, as dynamic and interconnected. Therefore, we recommend that interventions addressing such barriers involve different actors (e.g., clients and providers) address two or more domains. The fact that we observed similar sets of barriers across service settings in both decades of research suggests that very little change has occurred in the client’s relationships with provider, organization, and system in the last two decades. This suggests the need for more systemic interventions focusing on the intersection of diverse stakeholders.

Our relational framework also challenges narratives attributing client’s limited access and adherence to integrated HIV and SUD services as being client-level issues. Research shows that injection drug users and other SUD clients can successfully undergo HIV treatment [62]. Notwithstanding, our review suggests that structural inequalities (e.g., lack of transportation and social resources) are at the root of clients’ unstable and often chaotic lives. These structural problems thus put clients at a disadvantage and render them unable to access services. Krüsi and colleagues [16] point out the overestimation of individual/behavioral-level variables and underestimation of social and structural factors in the extant literature. This perception appears also to influence providers to perceive their clients as lacking the capacity to acknowledge their problems, initiate services, or adhere to treatments [10, 42, 46]. However, here we do not intend to blame providers or to imply that all providers are ignorant of the organizational- or structural-level barriers. We acknowledge the complexities in practice that providers often are limited in what they can do to help with structural barriers such as stigma and racism. Calling out the discrepancy in causal attributions is to highlight clients’ perspectives and their lived experiences as important considerations in service designs and implementations. Interventions aiming to address these issues will need to focus on structural factors in order to develop a base upon which clients can begin to address their individual-level problems.

Fragmented and competitive funding streams and limited political and technical supports can discourage organizations from addressing clients’ dual HIV and SUD issues comprehensively [29]. Integration of HIV and SUD services may be accomplished by facilitating providers’ collaborations within and beyond organizational boundaries. Regrettably, very few articles in this review provided replicable models or systems of service integration. Our review does highlight the need for greater interprofessional and interorganizational collaborations for promoting clients’ access to both HIV and SUD treatment services [62]. Some articles [44, 47, 59] reported the lack of collaboration and knowledge sharing among service providers. Therefore, we recommend that “collaboration”—the flow of knowledge and expertise across professional and organizational boundaries—be spelled out through institutional arrangements and structural supports. These would be reinforceable behaviors with the potential to facilitate effective communications, sharing responsibilities, and integration of services [7, 8, 62].

This review found that some HIV clinics are located in areas with a relatively lower volume of SUD clients. Providers in these settings, less exposed to clients with SUD, had limited knowledge and experiences that they can use to inform their work with dual-diagnosis clients. This lack of exposure and knowledge may be a contributor to providers’ biases and stereotypes about clients. Similarly, providers lacking expertise and training to mitigate this gap might experience more stress and concerns about their skillset that may exacerbate relational problems with their clients. We have not found empirical evidence among the articles in the review to confirm this position; however, it is worth noting one study that suggested providers’ mental health status and stressful life conditions as a potential barrier to access and delivery of services [43]. This highlights the need for further research in this area and intervention development to improve self-care among providers. Social support attained through interprofessional collaboration training is highly recommended.

## Conclusion

In this paper, we proposed and applied a client-centered relational framework in order to identify barriers to the integration of HIV and SUD services. This approach highlights the dynamic interactions between clients and providers, organizations, and systems from which they can access the services they need. Instead of examining barriers on one or another domain, we examined challenges that clients with HIV and SUDs need to face vis-à-vis service providers, organizations, and structures. This process highlighted not only the central role of clients but also the interconnectedness of barriers across different domains. The relational framework also helps service organizations and providers to identify areas for improvement. When zooming in on barriers in particular levels, it is easy to blame clients’ individual attributes and behaviors, but doing so may result in stigmatizing and unjust practices. By taking a client-centered view, the relational framework helps providers and organizational leaders to recognize gaps in services and identify ways to address barriers to *integration* and not in isolation. This integrated approach might include training providers and managers in interprofessional and interorganizational collaboration.

## Data Availability

The data supporting the conclusions of this article are all published academic journal articles. They are available in Web of Science, EBSCO*host*, and PubMed.
